# Integrated Quantitative Proteomics and Metabolome Profiling Reveal MSMEG_6171 Overexpression Perturbing Lipid Metabolism of *Mycobacterium smegmatis* Leading to Increased Vancomycin Resistance

**DOI:** 10.3389/fmicb.2020.01572

**Published:** 2020-07-24

**Authors:** Zhuhua Wu, Wenjing Wei, Ying Zhou, Huixin Guo, Jiao Zhao, Qinghua Liao, Liang Chen, Xiaoli Zhang, Lin Zhou

**Affiliations:** ^1^Key Laboratory of Translational Medicine of Guangdong, Center for Tuberculosis Control of Guangdong Province, Guangzhou, China; ^2^School of Stomatology and Medicine, Foshan University, Foshan, China; ^3^School of Medicine, Jinan University, Guangzhou, China

**Keywords:** MSMEG_6171, lipid metabolism, *Mycobacterium smegmatis*, drug resistance, multi-omics

## Abstract

In recent years, the treatment of tuberculosis is once again facing a severe situation because the existing antituberculosis drugs have become weaker and weaker with the emergence of drug-resistant *Mycobacterium tuberculosis* (Mtb). The studies of cell division and cell cycle-related factors in Mtb are particularly important for the development of new drugs with broad-spectrum effects. *Mycobacterium smegmatis* (Msm) has been used as a model organism to study the molecular, physiological, and drug-resistant mechanisms of Mtb. Bioinformatics analysis has predicted that MSMEG_6171 is a MinD-like protein of the septum site-determining protein family associated with cell division in *Mycobacterium smegmatis*. In our study, we use ultrastructural analysis, proteomics, metabolomics, and molecular biology techniques to comprehensively investigate the function of MSMEG_6171. Overexpression of MSMEG_6171 in Msm resulted in elongated cells, suggesting an important role of MSMEG_6171 in regulating cell wall morphology. The MSMEG_6171 overexpression could enhance the bacterial resistance to vancomycin, ethionamide, meropenem, and cefamandole. The MSMEG_6171 overexpression could alter the lipid metabolism of Msm to cause the changes on cellular biofilm property and function, which enhances bacterial resistance to antibiotics targeting cell wall synthesis. MSMEG_6171 could also induce the glyceride and phospholipid alteration *in vivo* to exhibit the pleiotropic phenotypes and various cellular responses. The results showed that amino acid R249 in MSMEG_6171 was a key site that can affect the level of bacterial drug resistance, suggesting that ATPase activity is required for function.

## Introduction

Mycobacterial diseases are a major public health concern caused by mycobacteria species including TB, leprosy, and atypical mycobacterial infections (Russell et al., 2010; Tiwari et al., 2018; Katale et al., 2019). The TB treatment faces a serious situation because of the multidrug-resistant tuberculosis (MDR-TB) (Pontali et al., 2017). The atypical mycobacterial infections are mainly caused by opportunistic pathogens from non-tuberculous mycobacteria. Non-tuberculous mycobacteria are inherently resistant to most commonly used antimicrobials and many anti-TB drugs (Falkinham, 2018). Thus, it is urgent to eradicate pathogenic mycobacteria from infection sources and hosts.

Mycobacterial species, especially Mtb, have a characteristic lipid-rich (up to 60%) cell wall that confers low permeability to many common antibiotics and chemotherapeutic agents (Bhat et al., 2017). The cell wall lipids also allow proliferation in host by inducing the formation of foamy macrophages (Santucci et al., 2016). Many antimicrobial drugs target this well-organized barrier of mycobacterial cell envelope (Rombouts et al., 2012; Lee et al., 2017; Rodriguez-Rivera et al., 2018). This well-organized barrier is composed of plasma membrane, cell wall core, and outermost layer (Hett and Rubin, 2008; Grzegorzewicz et al., 2016). The plasma membrane forms the bulk of the inner leaflet (Brennan, 2003). A variety of soluble lipids and proteins make up the outermost layer, including lipids with some long- and short-chain fatty acids complementing each other (Kaur and Kaur, 2017). The cell wall core is composed of peptidoglycan, which is covalently linked to heteropolysaccharide arabinogalactan via P-GlcNAc-Rha. A large variety of unusual lipids in the formation of cell walls are thought to enhance cell wall impermeability and to increase the intrinsic resistance of mycobacteria to antimicrobial drugs (Rens et al., 2016). Enzymes involved in lipid biosynthesis, intracellular transport, and utilization become the excellent therapeutic intervention targets (Bailo et al., 2015). Recent studies have shown that an increasing number of genes are involved in lipid synthesis, modification, and transport of cell wall components (Ripoll et al., 2007; Pang et al., 2013; Grzegorzewicz et al., 2015; Aguilar-Ayala et al., 2017).

*Mycobacterium smegmatis* (Msm) shares more than 2000 homologous genes with Mtb (Li et al., 2016). The unique Msm cell wall structure is similar to that of Mtb and many other mycobacterial species (King, 2003). Therefore, Msm is often used to study the molecular, physiological, and drug resistance mechanisms of Mtb. MSMEG_6171 is an uncharacterized protein in Msm; although it is similar to the MinD-like protein, it belongs to the MinD/Ssd family (England et al., 2011). Previous studies have shown that MinD and MinC jointly constitute the conservative center of Min system (Conti et al., 2015). Through recruiting MinC to the cell membrane, MinD can interact with MinC to inhibit the polymerization of FtsZ, thus restricting the assembly of Z-ring to the middle of cells (Lutkenhaus and Sundaramoorthy, 2003; Conti et al., 2015; Schuhmacher et al., 2015). The homolog of MSMEG_6171 in Mtb, Rv3660c, shares 61% amino acid sequence identity, and it characterizes as a putative septum site determining protein eliciting bacterial filamentation (England et al., 2011). It also elicits responses including the dormancy regulon and alternative sigma factors involved in adaptive metabolism (England et al., 2011). These conclusions are based primarily on transcriptome data and bioinformatic prediction without sufficient experimental data. Therefore, we undertook a study to experimentally determine the function of MSMEG-6171.

Here, we present a thorough investigation of MSMEG_6171 in Msm. First, we found that MSMEG_6171 overexpression can affect the bacterial biofilm properties and can resist the antibiotics targeting the bacterial cell wall. Secondly, our results showed that MSMEG_6171 can induce the glyceride and phospholipid alteration to display the pleiotropic phenotypes and various cellular responses *in vivo*. Thirdly, MSMEG_6171 overexpression can enhance the bacterial resistance to some antibiotics involved in cell wall synthesis. Finally, we also found that the amino acid R249 in MSMEG_6171 was a key site that can affect the level of bacterial resistance. Taken together, we proved that MSMEG_6171 overexpression can alter the lipid metabolism of Msm to change the structure and function of the mycobacterial cell envelope and to enhance bacterial resistance to antibiotics.

## Materials and Methods

### Bacterial Growth and Plasmid Constructs

Msm and its recombinant strains were cultured as described by Parish et al. (2001). A *Mycobacterium smegmatis* mc^2^155 mutant without *msmeg_6171* was constructed by performing allelic exchange using the *p1NIL/pGOAL19*-based flexible cassette (Parish and Stoker, 2000). Briefly, the 855-bp upstream and 849-bp downstream sequences of the *msmeg_6171* gene were amplified using oligonucleotides 6171UF/6171UR and 6171DF/6171DR. *Msm:6171*, NTD, and CTD of *msmeg_6171* were constructed by subcloning the *Eco*RI-*Hin*dIII fragment from the Msm genome into the corresponding sites in the *pMV261* vector. *msmeg_6171* was mutated by the site-directed mutagenesis method using three pairs of specific primers, respectively (Supplementary Table S1). The eGFP gene was cloned into *pMV261-MSMEG_6171* to generate the plasmid *pMV261-MSMEG_6171-eGFP* (see Supplementary Materials and Methods). All bacterial strains and PCR oligonucleotides used in this study are listed in Supplementary Table S1.

### Total RNA Isolation and Handling, and RT-qPCR Analysis

The total RNA of *Mycobacterium smegmatis* mc^2^155 was extracted and converted to cDNA using commercial kits (FastRNA^TM^ Pro Blue Kit, MP Biomedicals, United States; SuperScript III First-Strand Synthesis Kit, Thermo Fisher Scientific, United States). The mRNA expression of 7 genes (*msmeg_6171*, *mps2*, *msmeg_0400*, *msmeg_4757*, *msmeg_6761*, *pks*, *msmeg_3375*) were analyzed using SYBR Green (Maeda et al., 2003). SigA served as the internal reference. Mean ± SEM was calculated from three independent experiments, and significant difference analysis was determined by Student’s unpaired *t*-test.

### Phenotypic Analysis

Msm and its mutant strains were used for growth curve analysis, sliding motility assay, Congo Red assay, and biofilm assay. Bacteria culture were adjusted to an optical density at 600 nm (OD_600_) of 0.2, and growth curves were obtained from OD_600_ detected at intervals of 3 h. 2 μL culture of each bacterial strain was dispensed on the medium (LB medium supplemented with 100 μg/mL Congo Red (Sigma) and 20 μg/ml kanamycin, Sauton liquid medium, and 7H9 medium supplemented with 0.3% agar) and then was used for corresponding Congo Red assay, biofilm assay, and sliding motility assay (Zanfardino et al., 2016; Lai et al., 2018). Cells were incubated at 37°C for 3 days.

### Scanning Electron Microscopy (SEM)

Scanning electron microscopy was performed as described previously (Dahl, 2004). Msm strains were grown to stationary phase. Culture aliquots were concentrated by centrifugation, resuspended, and fixed with 2.5% glutaraldehyde. Then, sequential ethanol dehydrations were performed. After permutation with isopentylosacetate, critical point drying was determined using a Hitachi HCP-2 Critical Point Dryer (Hitachi High-Technologies Corp., Tokyo, Japan). Samples were gold sputter coated (Technics Hummer V; Anatech), and ultrastructure examination was performed using a Zeiss Ultra 55 electron microscope.

### Transmission Electron Microscopy (TEM)

Cells were processed for TEM as described previously (Vijay et al., 2012). In brief, Msm strains were harvested by centrifugation and fixed in 1% (vol/vol) osmium tetroxide (Sigma-Aldrich) and 0.15 M sodium cacodylate buffer (pH 7.2) (Sigma-Aldrich) for 1 h at room temperature and postfixed for 2 h at room temperature in 0.15 M cacodylate buffer (pH 7.2) containing 2% (wt/vol) tannic acid and 2% (vol/vol) glutaraldehyde. Samples were dehydrated in a graded ethanol series and embedded in Spurr resin. Thin sections (70 nm) were cut and stained with 1% uranyl acetate and Reynold’s lead citrate and observed under a Tecnai Spirit (FEI) transmission electron microscope at 100 kV. Images were captured at a magnification of 98000×; analyses of cell wall thickness were performed using ImageJ.

### Drug Susceptibility Testing (MIC)

The standard broth dilution method (Mengatto et al., 2006) was used to determine the MICs of Msm strains to different antibiotics (isoniazid, rifampicin, streptomycin, vancomycin, ethionamide, cycloserine, meropenem, cefoxitin, cefamandole). MICs were determined at 2 or 3 days post-incubation. Three independent experiments were performed. The detailed description is shown in Supplementary Materials and Methods.

### FLAG-Based Pull-Down Assay

Proteins interacting with MSMEG_6171 were analyzed by Flag-based pull-down assay according to previously published procedures with some modifications (Meniche et al., 2014). The protein complexes were separated by SDS-PAGE, visualized with silver staining, and then submitted to LC-MS analysis (see Supplementary Materials and Methods).

### Proteomics Analysis

Proteins were extracted, digested, and labeled using the iTRAQ(R) Reagents Multiplex Kit (sigma) following the manufacturer’s instructions. Chromatography–mass spectrometry (LC-MS) and bioinformatics analysis were performed according to previously published procedures with some modifications (Sharma et al., 2015). Three biological replicates were provided for each group (see Supplementary Materials and Methods).

### Yeast Two-Hybrid (Y2H) Assay to Detect MSMEG_6171-Interacting Proteins

The MSMEG_6171-interacting prey proteins Were cloned in *pGADT7AD*, and the gene for the bait protein MSMEG_6171 Was cloned Into *pGBKT7* (Matchmaker Gold Yeast Two-Hybrid System, Clontech, United States) to identify MSMEG_617–protein interactions according to the manufacturer’s instructions (Fields and Song, 1989).

### TLC and ESI/MS Analysis of Fatty Acids

Extraction and purification of fatty acids were performed as previously described (Liu and Nikaido, 1999). Cells were cultured and pelleted by centrifugation and resuspended in a saponification mixture (5% KOH in 88% methyl cellosolve), and the mixture was heated at 121°C for 1 h and cooled to room temperature. The mixture was acidified by adding 1.5 ml of 6 N HCl and was extracted with CHCl_3_. The CHCl_3_ layer was transferred into a glass tube, and the solvent was evaporated in a water bath at 80°C. The fatty acids were determined by analytical TLC on Silica Gel 60 (Macherey-Nagel) using dichloromethane/hexane (3:1; v/v) and revealed by spraying the plates with molybdophosphoric acid (10% in ethanol). Fatty acid samples were dissolved in diethyl ether and subjected to ESI/MS analysis in the negative ion mode. Mass spectra were determined on an Agilent 6500 Series Q-TOF B.08.00 (B8058.3SP1) mass spectrometer equipped with an ESI source. Data acquisition and analysis were performed using Qualitative Navigator B.08.00 software.

### Metabolome Assay

Bacterial strains were centrifuged, washed, and stored at −80°C for subsequent extraction of metabolites. For intracellular metabolites, samples were reconstituted and disrupted by FastPrep-24 (MP Biomedicals) (Carette et al., 2018). The intracellular metabolites were analyzed in the positive ion mode on an AB 5600 + TripleTOF mass spectrometer system. The relevant parameters were referred to previous studies (Khan et al., 2017). MS-DIAL version 2.24 and MONA were used for further data process and analysis (Horai et al., 2010). For lipid extraction, the chloroform/methanol solution (2:1, v:v) was added, mixed, and carried out by an ultrasonic cleaner. The supernatant was dried by N_2_ and then was dissolved in chloroform/methanol solution (2:1, v:v). For lipid samples, Dionex UltiMate 3000 (UHPLC)–Thermo Orbitrap Elite was used for LC-MS analysis. The chromatographic column used was Waters UPLC BEH C18 (1.7 μm 100^∗^2.1 mm). Mass spectrometry was operated in both positive and negative ion modes with the parameters optimized according to previously published procedures with some modifications (Layre et al., 2014). Raw data were converted to mz. data format by Agilent MassHunter Qualitative Analysis B.08.00 software (Agilent Technologies, United States). The XCMS program was used to further process and analyze the data. Visualization matrices containing the sample name, m/z-RT pair, and peak area were obtained after the internal standard normalization and weight normalization. The data matrices were imported into SIMCA-P 14.0 (Bjerrum et al., 2015) (Umetrics, Umea, Sweden), mean-centered, and scaled to Pareto variance. MetaboAnalyst software was used for multidimensional statistical analysis. The significantly different metabolites were determined with variable influence of *P*-values less than 0.05.

## Results

### MSMEG_6171 Overexpression Can Affect the Morphology of *Mycobacterium smegmatis*

**FIGURE 1:**
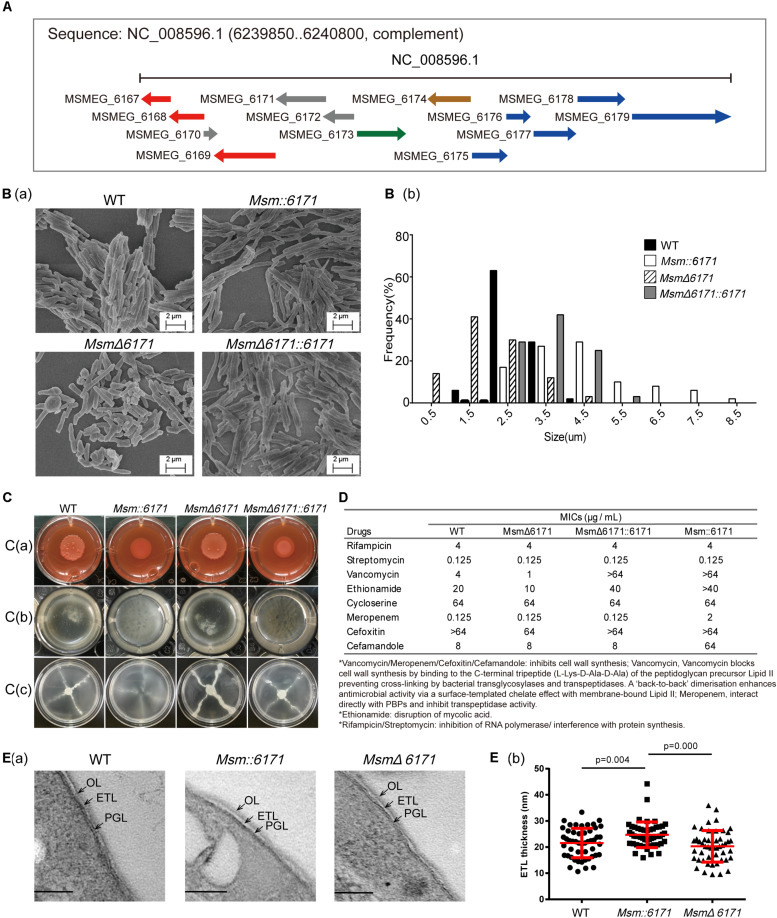
Phenotypic analysis of recombination Msm strains. **(A)** Schematic representation of the *msmeg_6171* gene cluster and biosynthetic locus on the chromosome. The genes involved in the secretion system of cell membranes (red), morphological differentiation (green), and metabolism (blue) are indicated. **(B)** Msm strains were visualized by scanning electron microscopy (SEM). **(B)(a)** Images are representative of different fields of bacteria from exponentially growing cultures at 37°C. **(B)(b)** Lengths of the bacterial cells were calculated from the coordinates of both ends of the cell as measured from representative fields as visualized by scanning electron microscopy. Multiple fields were examined and values calculated in 0.5–1-mm increments from multiple fields of over 100 cells. **(C)** Compared with the WT strain, *Msm:6171*, *Msm*Δ*6171*, and complemented (*Msm*Δ*6171:6171*) strains were tested with the following phenotypes: **(C)(a)** Colony morphology on Congo Red, **(C)(b)** biofilm production at the air–liquid interface, and **(C)(c)** sliding motility. **(D)** Drug susceptibility of *Msm:6171*, *Msm*Δ*6171*, and *Msm*Δ*6171:6171* compared with WT strains. ^∗^MIC in μg/mL. **(E)** Ultrastructures of cell envelopes of Msm. **(E)(a)** TEM images of Msm cells with detailed ultrastructure of cell envelope layers OL, ETL, and PGL. OL, outer layer; ETL, electron transparent layer; PGL, peptidoglycan layer. **(B)(b)** Cell envelope ETL thickness measurement between WT, *Msm:6171*, and *Msm*Δ*6171*. 50 cells of each strain were measured. Scale bar = 100 nm.

Bioinformatics analysis indicated that MSMEG_6171 is a MinD-like protein, and its adjacent proteins are involved in the secretion system of cell membranes, morphological differentiation, and metabolism (Figure 1A). To experimentally evaluate the physiological role of MSMEG_6171 in Msm, we constructed a recombinant *msmeg_6171* overexpression strain (*Msm:6171*), a *msmeg_6171* deleted strain (*Msm*Δ*6171*), and an *Msm*Δ*6171* strain complemented with *msmeg_6171* (*Msm*Δ*6171:6171*) (Supplementary Figure S1). Consistent with the previous study of Ssd protein in Mtb (England et al., 2011), *Msm:6171* strains were also observed with significantly increased cell lengths (3.6 ± 0.42 μm) and *Msm*Δ*6171* were significantly decreased (1.35 ± 0.51 μm), compared to wild-type strains (2.50 ± 0.11 μm). We also found that the cell lengths were partly recovered in the compensation strains (3.30 ± 0.42 μm) (Figure 1B). The viability of *Msm*Δ*6171* strains showed that MSMEG_6171 was non-essential for the Msm culture *in vitro*. Using Vancomycin-BODIPY assay, we did not observe any inhibition or enhancement of the septum formation indicating that the filamentation may not relate to suppression or enhancement of septum formation in the merodiploid and null strains (Supplementary Figure S2).

Interestingly, overexpression of MSMEG_6171 resulted in a drastic change in the colony morphology from a rough to a smooth phenotype on solid medium [Figure 1C(a)] and exhibited an altered capacity to assemble into biofilms [Figure 1C(b)]. In addition, the *Msm:6171* strains also showed a significantly increased sliding motility [Figure 1C(c)]. This phenomenon may be the result of the highly hydrophobic surface of the mutant cells, which plays an important role in the sliding motility (Maya-Hoyos et al., 2015). The MSMEG_6171-GFP fusion protein was constructed and introduced into *MsmΔ6171* strains to detect the localization of MSMEG_6171 in Msm. Fluorescence microscopy analysis showed that the fluorescence signal was detected in the cytosol, indicating the probable localization of MSMEG_6171 (Supplementary Figure S3). The subcellular localization of MinD to division sites reported by Marston et al. (1998) showed similar results: Polar localization was not observed as seen in *B. subtilis*.

To investigate whether MSMEG_6171 could affect the drug susceptibility of Msm, we measured the minimum inhibitory concentration (MIC) of eight different antibiotics (rifampicin, streptomycin, vancomycin, ethionamide, cycloserine, meropenem, cefoxitin, and cefamandole) with the four strains. For all the strains, we found that the MICs of rifampicin, streptomycin, and cycloserine were at the same level. Notably, the *Msm:6171* recombinant strains were 16-fold more resistant to vancomycin than the WT strains. Consistently, *MSM*Δ*6171* strains were more susceptible to vancomycin. A similar phenomenon was obtained using ethionamide. In addition, the *MSM:6171* strains had significantly increased resistance to meropenem and cefamandole (Figure 1D). Since vancomycin, meropenem, or cefamandole can inhibit cell wall synthesis, ethionamide is involved in disruption of mycolic acid; these data revealed that dramatic changes in the properties of the cell envelope in *Msm:6171* recombinant strains could affect the integrity and permeability of the cell envelope, as well as the organization and motility of the bacterial populations. Additionally, the null strains had little change on the envelope integrity and permeability, while *Msm*Δ*6171:6171* generally resulted in intermediate phenotypes, indicating that these phenomena are directly related to overexpression of MSMEG_6171.

To further investigate the effects of MSMEG_6171 on bacteria at the cell envelope, we investigated cell envelope ultrastructural features in WT and recombinant strains by transmission electron microscopy. The ultrastructure of these cells displayed a triple-layered cell envelope which are composed of the electron dense outer layer (OL), electron transparent layer (ETL), and peptidoglycan layer (PGL). The ETL of the cell envelope had an average thickness of 24.70 nm (±4.91 nm) in *Msm:6171* and 21.57 nm (±5.61 nm) in WT) (Figure 1E).

### MSMEG_6171 Overexpression Perturbs Lipid Biosynthesis and Metabolism to Alter the Cell Envelope Properties in *Mycobacterium smegmatis*

**FIGURE 2:**
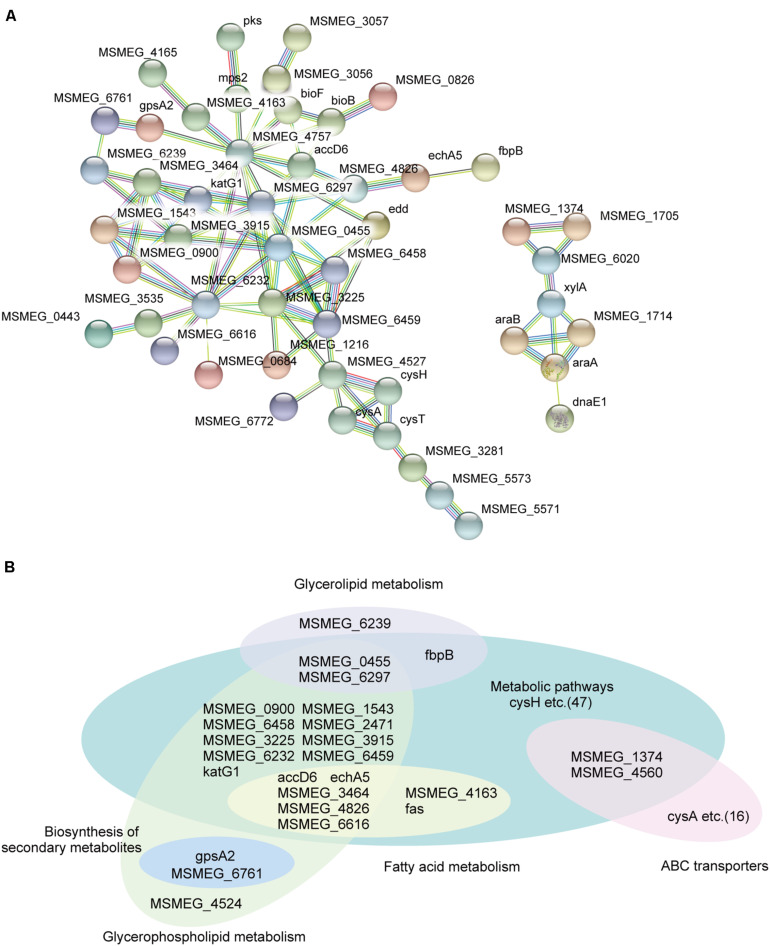
Network analysis of proteins potentially regulated by MSMEG_6171. **(A)** A subset of candidate MSMEG_6171-regulated proteins involved in the signaling pathways of the metabolic pathways and ABC transporters. **(B)** Classification of candidate MSMEG_6171 regulated proteins, including biosynthesis of secondary metabolites, fatty acid metabolism, glycerolipid metabolism, and glycerophospholipid metabolism.

To further gain new insights into how MSMEG_6171 affects bacterial cell envelope properties, we performed global protein expression profiling on the *Msm:6171* strains, *Msm*Δ*6171* strains, and the WT strains. Compared to the WT strains, 440 proteins were differentially expressed with fold changes of 1.5 or more (*p* values < 0.05), including 279 upregulated proteins and 161 downregulated proteins in the *Msm:6171* strains. In the *Msm*Δ*6171* strains, 70 downregulated proteins and 126 upregulated proteins exhibited fold changes greater than 1.5 (Supplementary Table S2). Gene Ontology (GO) enrichment analysis and KEGG pathway analysis were also performed to classify the function of those significantly altered proteins. We conducted a protein–protein interaction network analysis for proteins encoded by those potential MSMEG_6171 target genes using STRING 10.014. Among the upregulated proteins in the *MSM:6171* strains, we found that a large number of candidates, MSMEG_6171-regulated proteins, are involved in glycerolipid metabolism, fatty acid metabolism, glycerophospholipid metabolism, and ABC transporter (Figures 2A,B). Interestingly, among the downregulated proteins in the *MSM:6171* strains, 22 ribosomal proteins and 19 proteins associated with amino acid metabolism were enriched, which may be related to the slower growth of *MSM:6171* strains (Supplementary Figure S4).

**FIGURE 3:**
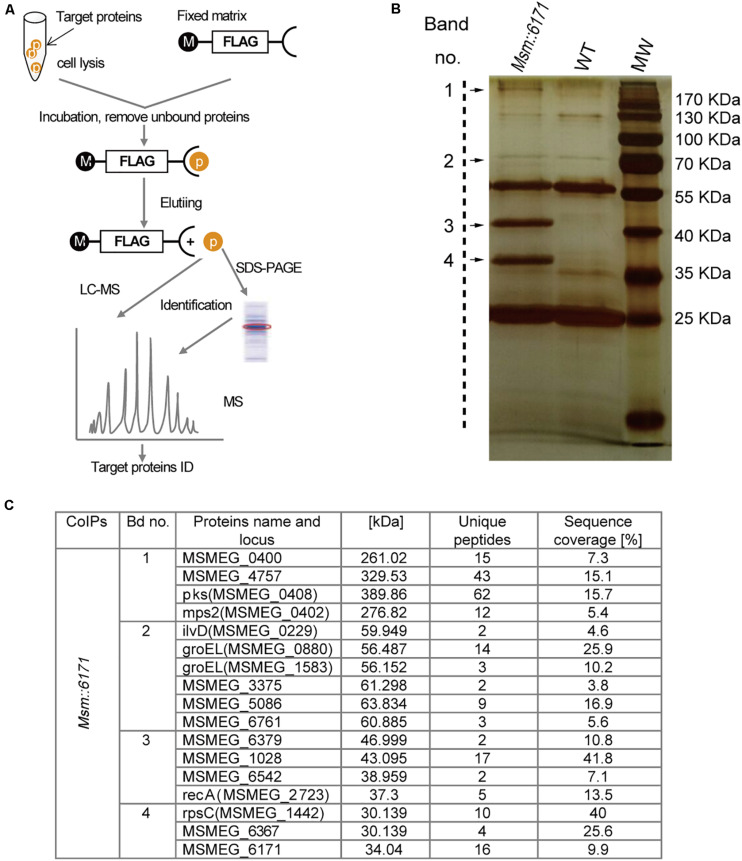
MSMEG_6171 interacts with proteins involved in lipid synthesis and metabolism. **(A)** FLAG-based pull-down scheme. Pull-down assay was performed with anti-FLAG M2 affinity gel, and the eluate was separated by SDS PAGE, and visualized with silver staining. **(B)** Then, the target bands were excised and subjected to in-gel tryptic digestion for LC-MS analysis. Tandem mass spectrometry of specific proteins in the target bands were shown in **(C)**. WT, wild-type Msm mc^2^155 strains; MW, molecular weight standards.

**FIGURE 4:**
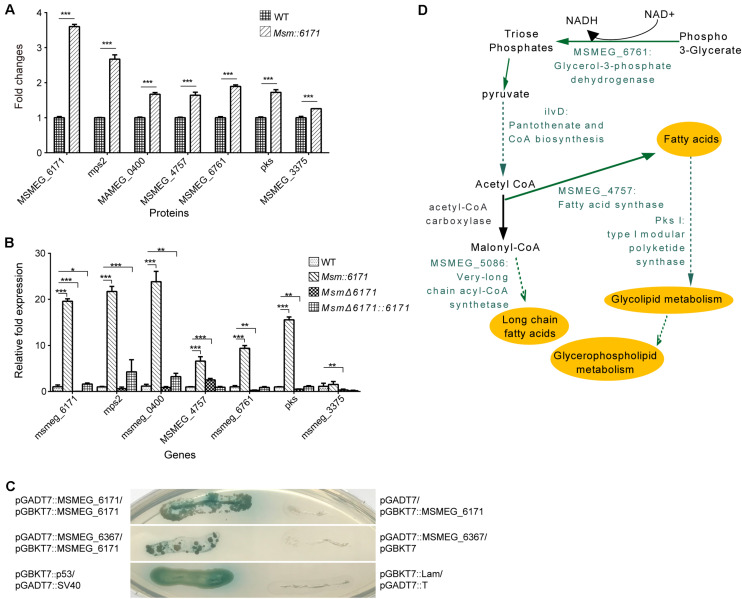
Identification of proteins interacting with MSMEG_6171. **(A)** Fold change of protein expression levels in *Msm:6171* (including MSMEG_6171, mps2, MSMEG_0400, MSMEG_4757, MSMEG_6761, pks, MSMEG_3375) were calculated relative to WT control. ^∗∗∗^ represents *P*-value < 0.01. **(B)** Semi-quantitative RT-PCR analysis was performed to verify the expression of genes corresponding to the proteins in Figure 3C. Data were analyzed using the ΔΔCT method with the *M. smegmatis* reference gene sigA as the control. Data were mean values ± SD from independent biological samples. ^∗∗∗^ represents *P*-value < 0.01, and ^∗^ represents *P*-value < 0.05. **(C)** The GAL4-based Matchmaker Gold Yeast Two-Hybrid system was used to identify interacting partners of MSMEG_6171, which was cloned into the pGBKT7 vector as a fusion to the GAL4 DNA-binding domain (pGBKT7:MSMEG_6171). The prey proteins of MSMEG_6171 in the pull-down assay were expressed as fusions to the Gal4 activation domain using pGADT7AD. The screen revealed that MSMEG_6171 interacts with MSMEG_6367 and itself. P53 and SV40 were exploited to serve as the positive control. pGBKT7:MSMEG_6171/pGADT7AD, pGBKT7/pGADT7AD:MSMEG_6367, and pGBKT7:Lam/pGADT7:T represent negative controls. **(D)** Schematic summary of the specific proteins involved in lipid metabolism pathways. The green characters represent the significantly upregulated candidate MSMEG_6171-regulated proteins.

We next focused on the proteins which can interact with MSMEG_6171 (Figure 3A). Seventeen proteins were found using pull-down assay (Figures 3B,C). Among these proteins, six proteins were significantly upregulated in the *Msm:6171* recombinant strains compared with WT (Figure 4A). Quantitative RT-PCR analysis was also performed to verify the differentially expressed proteins at the level of their mRNA (Figure 4B). The results showed that MSMEG_4757, Mps2, Pks, MSMEG_0400, and MSMEG_6761 could increase with the overexpression of MSMEG_6171, suggesting that these proteins could interact directly or indirectly with MSMEG_6171. Notably, these proteins were involved in the activation of the lipid synthesis and metabolism (Figure 4D), indicating that MSMEG_6171 may play a role in the synthesis and metabolism of the lipid portion of cell envelope in *Msm*. We next tried to verify *in vitro* which proteins can directly interact with MSMEG_6171. We expressed MSMEG_6171 in *Escherichia coli* (*E. coli*), but we found that *E. coli*-overexpressed MSMEG_6171 could not survive. It indicated that MSMEG_6171 is toxic to *E. coli*. Thus, it was difficult to determine whether these proteins directly interacted with MSMEG_6171 or not *in vitro*. Using the yeast two-hybrid screen with MSMEG_6171 as bait, we tested these proteins obtained from pull-down assay except MSMEG_4757, Mps2, Pks, and MSMEG_0400 which failed to construct into yeast. The screen identified MSMEG_6367, a galactofuranosyltransferase involved in cell wall biosynthesis, to be an interactor of MSMEG_6171 (Figure 4C). Besides, MSMEG_4757, Mps2, Pks, MSMEG_0400, and MSMEG_6761 showed no significant change in protein level in *MSM*Δ*6171* strains (Supplementary Table S2), although some corresponding mRNA decreased, such as pks and MSMEG_6761 (Figure 4B). As mentioned above, there were no significant differences in biofilm formation and Congo Red staining experiments between *Msm*Δ*6171* strains and WT strains. However, the *Msm:6171* strains became as smooth and moist as *E. coli* (Figure 1C). These phenotypic changes are most likely related to these upregulated proteins associated with the overexpression of MSMEG_6171. Based on those abovementioned data, we speculated that MSMEG_6171 may play a role in the lipid synthesis, metabolism, and biofilm formation in Msm.

### MSMEG_6171 Can Affect Lipid Metabolism in *Mycobacterium smegmatis*

To further investigate the effects of MSMEG_6171 on bacteria at the metabolomics level, the intracellular metabolites of *Msm:6171*, *Msm*Δ*6171*, and WT strains were extracted and determined by LC-MS under the same conditions to acquire the metabolomics profiles. To identify altered metabolites in *Msm:6171*, *Msm*Δ*6171* strains compared to WT strains, PLS-DA, and PCA was carried out for analysis. The score plot of the PCA obtained from the LC-MS data clearly discriminated between samples *Msm:6171* and WT strains, while not in *Msm*Δ*6171* (Supplementary Figures S5A,B). A total of 597 putative metabolites that were differentially expressed (*FDR* < 0.05) were identified in *Msm:6171* strains compared with the WT strains (Supplementary Table S3). Most of the lipids are glycerophospholipid and glycerolipid, viz. PA, PE, PC, PS, PI, PG and DG, MGDG, DGDG, and SQDG (Supplementary Figure S5C). Moreover, the changed metabolites mostly consisted of lipids and amino acids (Supplementary Figure S5D). KEGG pathway enrichment is shown in Supplementary Figure S5E, and glycerolipid metabolism and the glycerophospholipid metabolism pathway were the drastically altered metabolic activities through overexpression of MSMEG_6171 with considerable enrichment (*P* < *0.05*). Besides, some amino acids, such as phenylalanine, tyrosine, and tryptophan biosynthesis; glycine, serine, and threonine metabolism; phenylalanine metabolism; cysteine and methionine metabolism; and aminoacyl-tRNA biosynthesis, were also significantly enriched, as well as nucleotide-related pathways—purine metabolism and riboflavin metabolism.

**FIGURE 5:**
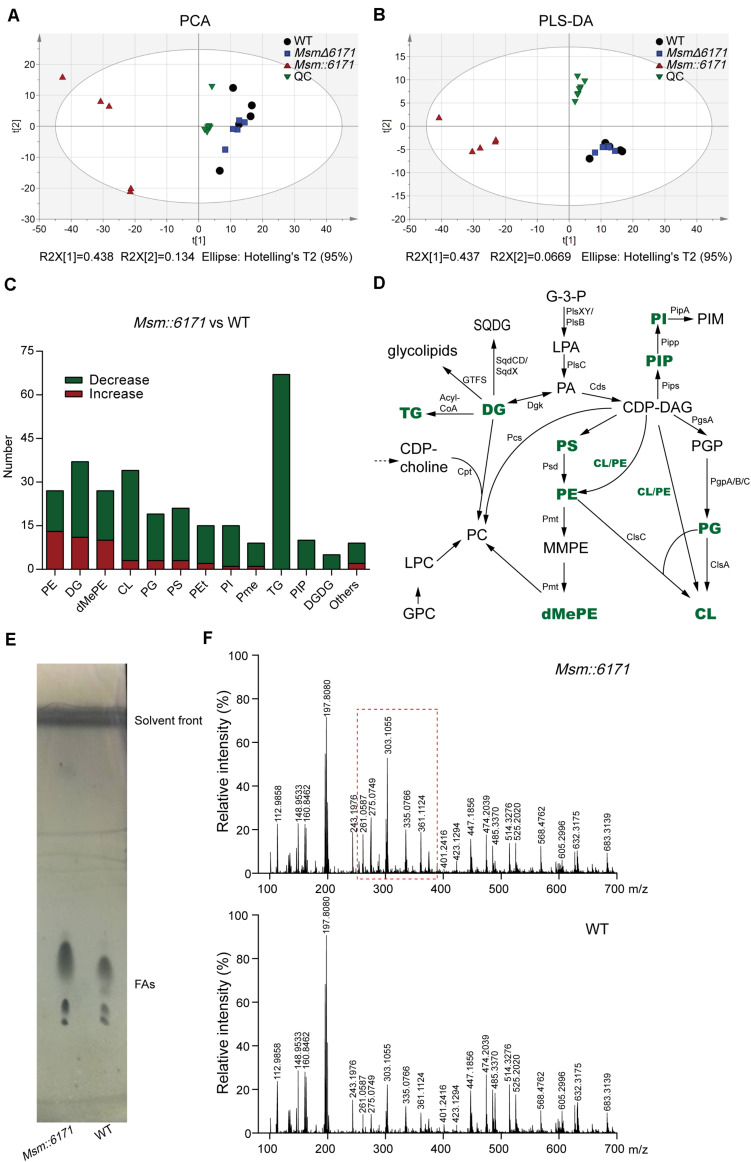
LC/MS-based metabolomics analysis of lipid metabolome. **(A)** PCA score and **(B)** PLS-DA score scatter plots acquired from LC-MS data in the ESI– and ESI+ modes. *R*^2^, the total explained variation of the model; *Q*^2^, the predictability of the model. WT, wild-type Msm mc^2^155 strain; QC, quality control. **(C)** The number of drastically altered lipids in the *Msm:6171* strains compared with the WT strains. Red, increased lipids; green, decreased lipids. **(D)** Schematic representation of enriched pathways involved in the synthesis of membrane lipids. Green characters represent the differentially expressed lipids identified in the *MSM:6171* strains. CL, cardiolipin; G3P, glycerol-3-phosphate; LPA, lysophosphatidic acid; PA, phosphatidic acid; GPC, glycerophosphocholine; PGP, phosphatidylglycerol phosphate; PG, phosphatidylglycerol; PIP, phosphatidylinositol phosphate; PI, phosphatidylinositol; PIM, phosphatidylinositol mannoside; SQDG, sulfoquinovosyl diacylglycerol; GTF, glycosyltransferase; DG, diacylglycerol; CDP, DG-cytidine diphosphate-diacylglycerol; PS, phosphatidylserine; MMPE, monomethyl PE; DMPE, dimethyl PE; PC, phosphatidylcholine; LPC, lysophosphatidylcholine; PE, phosphatidylethanolamine. **(E)** TLC analysis of fatty acids extracted from *Msm:6171* and WT. Visualization was by phosphomolybdic acid spray. **(F)** ESI-MS-TOF mass spectra analysis of organic extracts from *Msm:6171* and WT displaying the [M-H]^–^ ions.

The results of the above proteome and total metabolome showed that the function of MSMEG_6171 was related to lipid metabolism. Next, we conducted the lipid metabolome to further study the lipid composition with specific changes (Supplementary Table S3). The results of PLS-DA and PCA showed that the lipid profile of Msm:6171 could be clearly discriminated with WT strains, but *Msm*Δ*6171* did not show such difference (Figures 5A,B). We found that many lipids were significantly decreased in *Msm:6171* strains, which are major constituents of the cell membrane (Figure 5C). It suggested that there was a difference of the lipid membrane structure between the *Msm:6171* strain and WT strain. The significant changes of lipids were associated with the biosynthesis of lipoteichoic acid. For example, TGs, PGs, DGDGs, and DGs were drastically decreased, which could alter LTA biosynthesis in *Msm:6171* strains. Besides, the lipids with significant differences between the two groups were not only the precursor of LTA but also the main components of the Msm bacterial membrane. The balance of lipid components was critical for proper membrane–protein structure, transport function, DNA replication, and cell division. Changes in the composition of neutral lipids may disturb the permeability of the cell membranes by altering the surface charge of the membrane, such as TGs, DGDGs, and DGs, the major zwitterionic phospholipid PEs, and the major anionic phospholipids, phosphatidylglycerols (PGs) and CLs. Differentially expressed lipids were identified in the *MSM:6171* strains which were involved in the synthesis of membrane lipids (Figure 5D). Furthermore, we extracted and analyzed the fatty acids from the WT and *MSM:6171* strains to detect the change of fatty acids by TLC and ESI/MS. TLC analysis of fatty acid composition after saponification showed that these bands from WT and *MSM:6171* strains migrated at a similar rate, but the abundance of mycotic acid extracted from *MSM:6171* strains is higher than that of WT strains (Figure 5E). Consistently, ESI/MS profiles of free fatty acids of *MSM:6171* strains (Figure 5F) exhibited a relatively higher intensity of [M-H]^–^ ions ranging from *m/z* 261 to 361 than WT. In a nutshell, we speculated that MSMEG_6171 may play a role in the lipid synthesis, metabolism, and biofilm formation in Msm according to the above data.

### R249 Is an Important Active Cite for MSMEG_6171 to Improve the Resistance to Vancomycin

**FIGURE 6:**
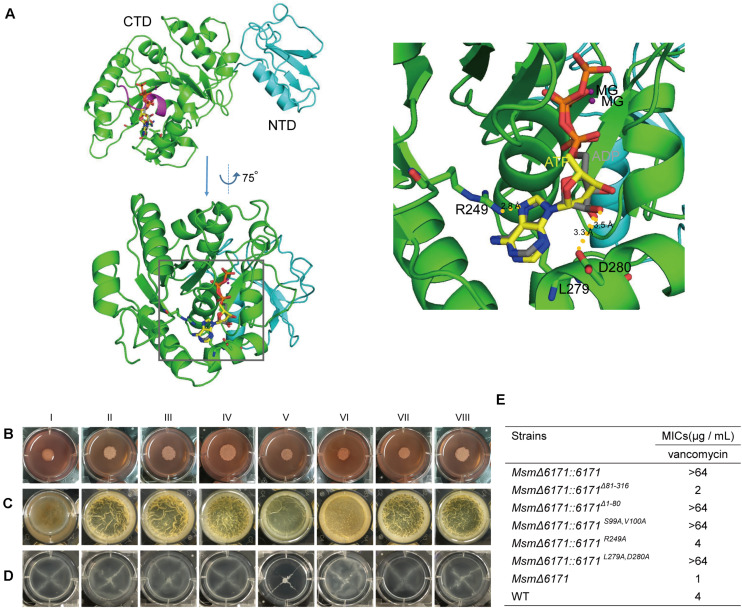
The *msmeg_6171* mutants can affect bacterial colony morphology, surface hydrophobicity, biofilm formation, and spreading. **(A)** 3D-structural model of MSMEG_6171 predicted by Phyre 2. The monomer of MSMEG_6171 consists of two domains: an N-terminal domain (NTD) (cyan cartoons) and a C-terminal domain (CTD) (green cartoons). The p-loop (^93^KGGAGASV^100^) is shown as a magenta cartoon, and the ligands (ATP and ADP) are shown as sticks. Interfaces between MSMEG_6171 and the ligands (ATP and ADP) predicted by Phyre 2. The ATP and ADP are shown as sticks, and the nucleic acid-binding pocket is marked with the black frame. (G) With enlargement of the region of the binding pocket, R249 interacts with the purine of ATP, and D280 shows the interaction with 2′-OH of ribose of ATP, and the side chain of L279 likes the scaffold to support the plane formed by the ribose of ATP. **(B)** Colony morphology on Congo Red, **(C)** biofilm formation, and **(D)** sliding motility. (I) *Msm*Δ*6171:6171*, (II) *Msm*Δ*6171:6171^Δ^^81– 316^*, (III) *Msm*Δ*6171:6171^Δ^^1– 80^*, (IV) *Msm*Δ*6171:6171^*S*99*A,V*100*A*^*, (V) *Msm*Δ*6171:6171^*R*249*A*^*, (VI) *Msm*Δ*6171:6171^*L*279*A, D*280*A*^*, (VII) *Msm*Δ*6171*, and (VIII) WT. **(E)** Analysis of the susceptibility of the *msmeg_6171* mutant strains to vancomycin.

Using Phyre2 analysis, we acquired a three-dimensional model of MSMEG_6171 and showed the potential binding sites of ligands (Mg^2+^, ATP, and ADP) (Figure 6A). MSMEG_6171 presented two domains, an NTD (from Met1 to Gly80) and a CTD (G81 to A316). The CTD of MSMEG_6171 is predicted to be an ATPase that is highly similar to the bacterial cell division-related ATPase MinD (PDB code: 1g3r,% i.d. = 19) of *Pyrococcus furiosus* and the chromosome segregation protein Soj (PDB code: 2bek,% i.d. = 23). In contrast to the canonical motif (GGxxGxGK[ST]), MinD/Soj-like ATPases contain a deviant Walker-A (p-loop) motif (xKGGxGK[ST]), with two lysines as a subgroup of ATPases. One of the lysines is near the carboxyl end of the motif, which is common to all Walker-A motifs. The other one is at the beginning of the motif (Lutkenhaus and Sundaramoorthy, 2003). The Walker-A motif of a pilus assembly protein, ErTadZ of *Eubacterium rectale*, is further degraded (^132^PCGGVGTS^139^) with the loss of both lysines but shows weak ATPase activity. The p-loop of MSMEG_6171 (^93^KGGAGASV^100^) contains an alanine at the position corresponding to the second lysine but has the first conserved lysine. All of the above findings suggest that MSMEG_6171 may be an ATPase that plays an important role in cell length in accordance with the phenotype of overexpression or knockout of *msmeg_6171*. In the nucleic acid-binding pocket of the model of MSMEG_6171, the conserved P-loop (^93^KGGAGASV^100^) is similar to a clamp holding the ATP or ADP molecule. R249 participates in a polar interaction with the purine of ATP. D280 interacts with the 2′-OH of the ribose of ATP. The side chain of L279 acts as a scaffold to support the plane formed by the ribose of ATP.

Based on the predicted results mentioned above, we constructed two deletion mutants (*Msm*Δ*6171:6171^Δ^^81–316^* and *Msm*Δ*6171:6171^Δ^^1–80^*) and three site-directed mutants of MSMEG_6171 (*Msm*Δ*6171:6171^*S*99*A,V*100*A*^*, *Msm*Δ*6171:6171^*R*249*A*^*, and *Msm*Δ*6171:6171^*L*279*A,D*280*A*^*) and then introduced these mutants into the *Msm*Δ*6171* strains to test the effects of the mutations on the bacterial cell surface and envelope properties *in vivo*. As shown in Figures 6B–D, we found that all the mutants especially *Msm*Δ*6171:6171^*R*249*A*^* exhibited significant changes in colony morphology, biofilm formation, and sliding motility compared to the WT strains, except for the mutant *Msm*Δ*6171:6171^*L*279*A,D*280*A*^*. Furthermore, the *Msm*Δ*6171:6171^Δ^^1–80^* and *Msm*Δ*6171:6171^*R*249*A*^* mutant strains were more susceptible to vancomycin than the WT strains (Figure 6E). Surprisingly, the resistance of Msm to vancomycin was significantly enhanced when only the CTD was expressed. The NTD was not sufficient for vancomycin resistance. Taken together, our results indicated that R249 plays an important role in the resistance to vancomycin.

## Discussion

Based on bioinformatics analysis, England et al. (2011) previously found that Rv3660c, the homologous protein of MSMEG_6171, plays an important role in cell morphology and adaptive metabolism. In this study, we experimentally characterized the role of MSMEG_6171 in Msm. Our results showed that the overexpression of MSMEG_6171 has an impact on bacterial physiology, from the increase in biofilm formation to the change in cell size and properties. These changes could alter the drug sensitivity of Msm to the antibiotics involved in cell wall synthesis, such as vancomycin. We found that filamentation possibly does not relate to Z-ring formation in the merodiploid and null strains of our experiments (Supplementary Figure S2). However, this result conflicted with England et al.’s (2011) previous report that the filamentation of Mtb *rv3660c* merodiploid strains had been caused by inhibition of FtsZ polymerization and Z-ring formation. Integrated proteome and metabolome results have shown that overexpression of MSMEG_6171 could regulate lipid biosynthesis and metabolism by inducing the alteration of glyceride and phospholipid to exhibit the pleiotropic phenotypes and various cellular responses. The results of this study enrich our understanding of the function of the MinD family proteins.

### Overexpression of MSMEG_6171 Can Regulate Lipid Biosynthesis and Metabolism of *Mycobacterium smegmatis*

A combination of Flag-based pull-down, proteomics, and bioinformatics analyses led us to discover many proteins involved in lipid biosynthesis and metabolism enriched in MSMEG_6171 merodiploid strains, such as MSMEG_4757, Mps2, Pks, MSMEG_0400, and MSMEG_6761. MSMEG_4757 is a fatty acid synthase (fas) that catalyzes fatty acid synthesis. MSMEG_6761, a glycerol-3-phosphate dehydrogenase (GPDH), can catalyze the reversible redox conversion of glycerone phosphate to sn-glycerol 3-phosphate (Ou et al., 2006). Pks belongs to a family of multidomain enzymes that produce polyketides as a category of lipids (Jenke-Kodama et al., 2005). The *mps2* gene encodes the mycobacterial peptide synthetase required for the formation of the tripeptide-amino-alcohol moiety, which is then linked to the 3-hydroxy/methoxy C_26_–C_34_ acyl chain, the lipid moiety of glycopeptidolipids (Jaspersen et al., 2006; Park et al., 2015). Our metabolomics results further showed that many glyceride and phospholipids were drastically decreased in merodiploid strains compared to wild strains and null strains, such as TGs, CLs, and other phospholipids. In Msm, the basic skeleton of the cell membrane showed the presence of glycerophospholipids including CL/PG, PE, PI, and PIMs. These glycerophospholipids isolated the cell from the external environment and ensured the independence of biochemical reactions as well as the formation of internal compartments in which different biological functions can be completed without interference (Pan et al., 2018).

It has been demonstrated that membrane lipid composition was considered to be a critical factor in determining cell size and shape, as well as a basic morphological parameter to ensure bacterial cell surface adhesion and biofilm formation. For example, the CL-lacking *E. coli* cells exhibit a filamentous phenotype in the M9 minimal medium. The elimination of CLs can result in a decrease in growth rate in the M9 medium, suggesting that CLs act as a crucial role in the maintenance of cell size and homeostasis and in proper cell division (Rowlett et al., 2017). A similar phenomenon emerged in this study that CL was significantly decreased when MSMEG_6171 was overexpressed, along with a lower growth rate and increased cell length. It is well known that DGs and TGs mainly exist in the outer membrane (mycomembrane) and inclusion bodies in the cytoplasm of Msm (Bansal-Mutalik and Nikaido, 2014). Baek et al. (2011) had demonstrated that TGs could play a causal role in governing growth, metabolic rate, and antibiotic susceptibility by reducing the carbon flux through the tricarboxylic acid cycle. Therefore, we hypothesized that the sharply reduced TG in MSMEG_6171-overexpressed strains was associated with bacterial phenotypic changes and drug sensitivity. The specific mechanism of its action required further study.

Notably, the significant changes in lipid profile and proteins associated with lipid biosynthesis and metabolism were only found in MSMEG_6171 merodiploid strains, not in *MsmΔ6171* strains. It indicated that MSMEG_6171 may not be a key regulatory protein involved in lipid metabolism. It is also possible that other proteins with similar functions can compensate for its function when MSMEG_6171 was deleted.

Our TEM result revealed that MSMEG_6171 alters envelope ultrastructure varies between *Msm:6171* and WT. The ETL is mainly composed of lipids like mycolic acids (Mdluli et al., 1998). Our proteomics and transcriptomics results showed that proteins involved in mycolic acid biosynthesis (fas) which interacts with MSMEG_6171 were significantly upregulated, and metabolomics results further showed that many glyceride and phospholipids were decreased in merodiploid strains compared to wild strains and null strains. All of these results indicate that active synthesis of mycolic acids may increase ETL thickness; consistently, TLC and ESI/MS analysis of mycolic acids is a reminder that MSMEG_6171 overexpression shows a positive correlation with mycolic acid content. Further investigations are needed to understand the role of MSMEG_6171 in regulating lipid synthesis and metabolism.

### Overexpression of MSMEG_6171 Increases Vancomycin Resistance of *Mycobacterium smegmatis*

Vancomycin is an antibiotic, which targets lipid biosynthesis by specifically binding to lipid II to hinder subsequent building blocks from the penicillin-binding proteins, thereby inhibiting transglycosylation and transpeptidation. We found that overexpression of MSMEG_6171 could decrease the susceptibility of Msm to vancomycin. The isogenic deletion of MSMEG_6171 increased the susceptibility to vancomycin. Furthermore, the expression of vancomycin-responsive genes, such as MSMEG_1764, MSMEG_3945, and MSMEG_0586 (Provvedi et al., 2009; Zeng et al., 2016) were decreased in MSMEG_6171 multiploid strains, following increased vancomycin resistance.

Some reports have also shown that the lipid composition of bacterial cells could correlate with antibiotic resistance, because they were involved in the biosynthesis of major membrane lipids and lipoteichoic acid. Hewelt-Belka et al. (2016) found that MGDG, PG, and DGDG were upregulated in antibiotic-resistant *S. aureus* strains, whereas the levels of DG lipid groups were downregulated. They also found that PG was increased while CL and Lys-PG levels were decreased in methicillin-sensitive strains, compared with methicillin-resistant *S. aureus*. Another study showed that the expression of plasmid-mediated resistance to fusidic acid in a *S. aureus* strain was associated with changes in the molar ratio of PG to lysl-PG (Chopra, 1976). Provvedi et al. (2009) also reported that treatment with 10X-MIC vancomycin induced strong repression of genes required for lipid metabolism (32%) in Mtb. Therefore, differences in the lipid patterns between MSMEG_6171 multiploid strains and wild strains suggested that vancomycin susceptibility may be associated with the lipid composition of bacterial cells.

In this study, through integrating scanning electron microscopy analysis, transmission electron microscopy, proteomics, metabolomics, and molecular biology techniques, we reveal that MSMEG_6171 could function in cell envelope organization via regulating the lipid metabolism of Msm; this may further enhance the bacterial antibiotic resistance and result in other different important cellular phenotypes. MSMEG_6171 is predicted to be a MinD/Soj-like ATPase by bioinformatics analysis. We also analyzed its ATPase active site and found that R249 could play an important role in the bacterial phenotype and vancomycin resistance. The ATPase activity of MSMEG_6171 and its regulations of drug sensitivity and lipid metabolism still need to be confirmed *in vitro* by substantial experiments.

## Data Availability Statement

The mass spectrometry proteomics data have been deposited to the ProteomeXchange Consortium via the PRIDE (Vizcaino et al., 2016) partner repository with the dataset identifier PXD014779 and 10.6019/PXD014779.

## Author Contributions

WW and ZW conceived, designed and supervised the overall study. XZ and LZ acquired funding and supervised and administered the project. WW and ZW provided the mutated mycobacterial strains. HG, QL, and LC coordinated the experiments to proceed smoothly. ZW, WW, YZ, and JZ processed the samples and performed the experiments. ZW, WW, and YZ analyzed the data. WW and ZW wrote the manuscript. All authors read and approved the final manuscript.

## Conflict of Interest

The authors declare that the research was conducted in the absence of any commercial or financial relationships that could be construed as a potential conflict of interest.

## Abbreviations

CL: cardiolipin
CTD: C-terminal domain
DG: diglyceride
DGDG: digalactosyl diglyceride
MGDG: monogalactosyl diglyceride
Msm: *Mycobacterium smegmatis*
Mtb: *Mycobacterium tuberculosis*
NTD: N-terminal domain
PA: phosphatidic acid
PC: phosphatidylcholine
PE: phosphatidylethanolamine
PG: phosphatidyl glycerin
PI: phosphatidylinositol
PIM: phosphatidyl-myo-inositol mannosides
pks: polyketide synthase
PS: phosphatidylserine
SQDG: sulfoquinovosyl diacylglycerol
TB: tuberculosis
TGs: triacylglycerols.
