# Supplementary material for: Integrated Quantitative Proteomics and Metabolome Profiling Reveal MSMEG_6171 Overexpression Perturbing Lipid Metabolism of Mycobacterium smegmatis Leading to Increased Vancomycin Resistance
Source: Front Microbiol. 2020 Jul 24;11:1572. doi: 10.3389/fmicb.2020.01572 (PMC7393984; doi:10.3389/fmicb.2020.01572)
Supplement: Supplementary file 1 [file Data_Sheet_1.PDF]

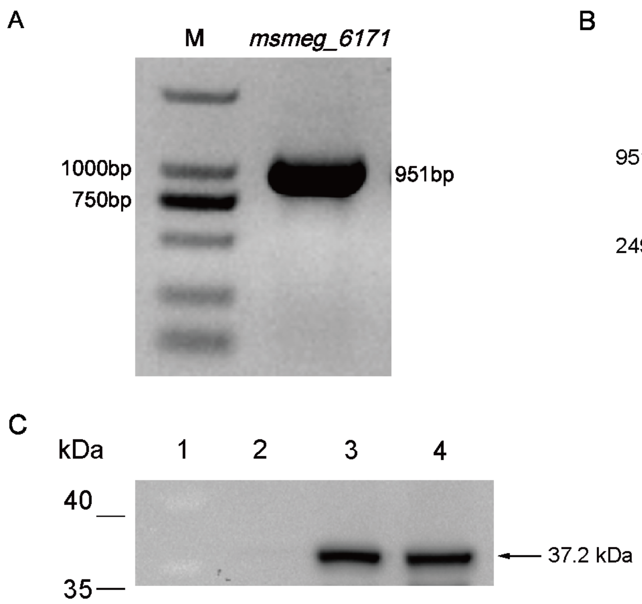

Supplemental Fig. S1. (A) The agarose gel electroporesis of PCR product of *msmeg_6171*. M: DNA ladder; **(B)** *msemg_6171* gene knockout strains *(MsmΔ6171)* was confirmed by PCR. M: DNA ladder; **(C)** Identification of MSMEG_6171 fusion protein expression by Western Blotting analysis. lane 1, prestained protein marker; lane 2, the total protein of wt strain; lane 3, the total protein of *Msm::6171* strain; lane 4, the total protein of *MsmΔ6171::6171* strain.

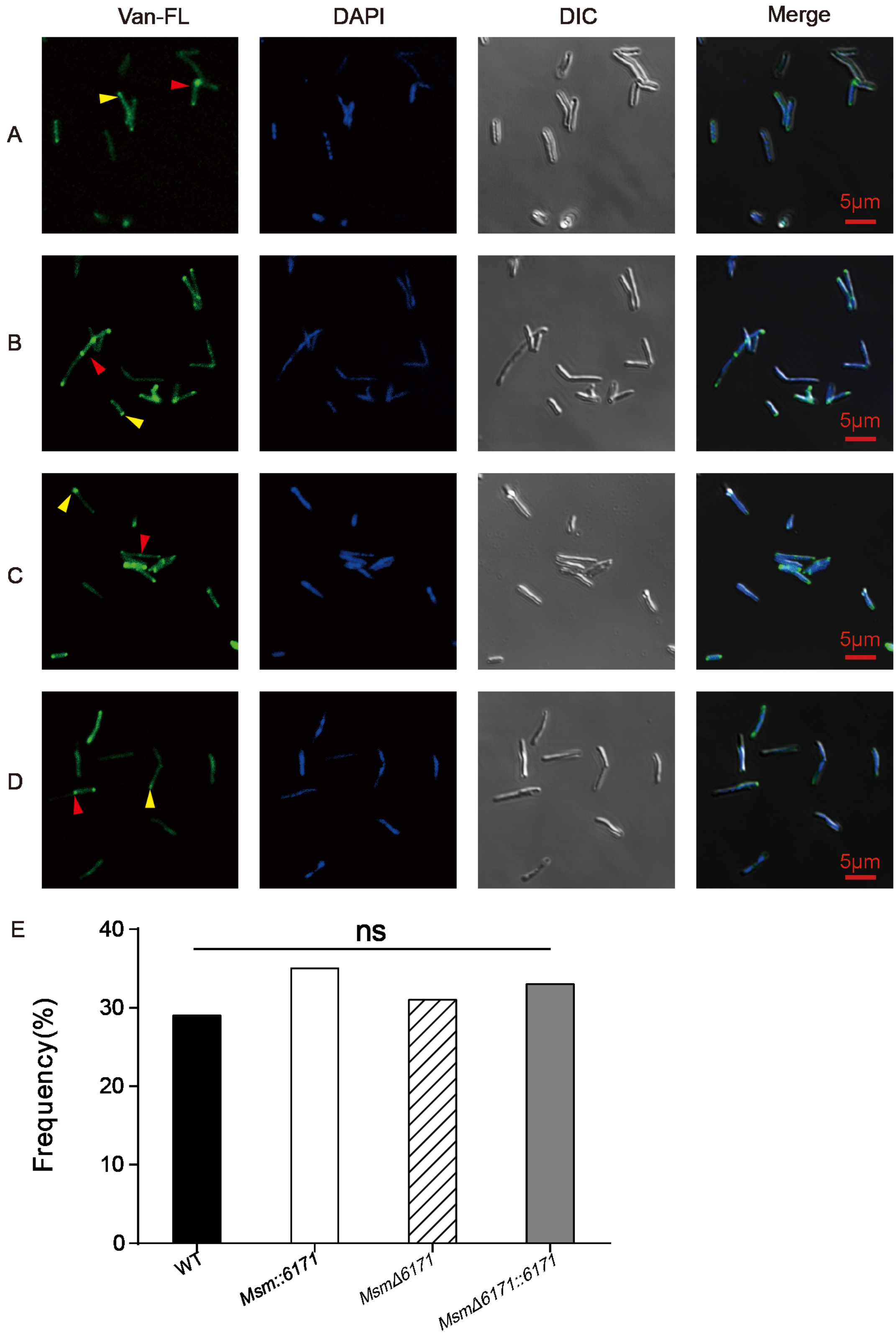

Supplemental Fig. S2. Van-FL staining and morphology of Msm mutant strains. For each strain micrographs of representative cells visualized by Van-FL staining (green) and DAPI staining (blue) are presented. Strains were examined using a Zeiss LSM880 inverted widefield micrcope and measured using Zen Blue 2.3 software. (A) WT; (B) *Msm::6171*; (C) *MsmΔ6171* and (D) *MsmΔ6171::6171*. Scale bars: 5 μm. yellow arrow:cell polar. red arrow: septum. (E) Data are represented as the frequency of cells with septum, n=100. No statistically (ns) significant differences were detected, comparing strains in each staining type.

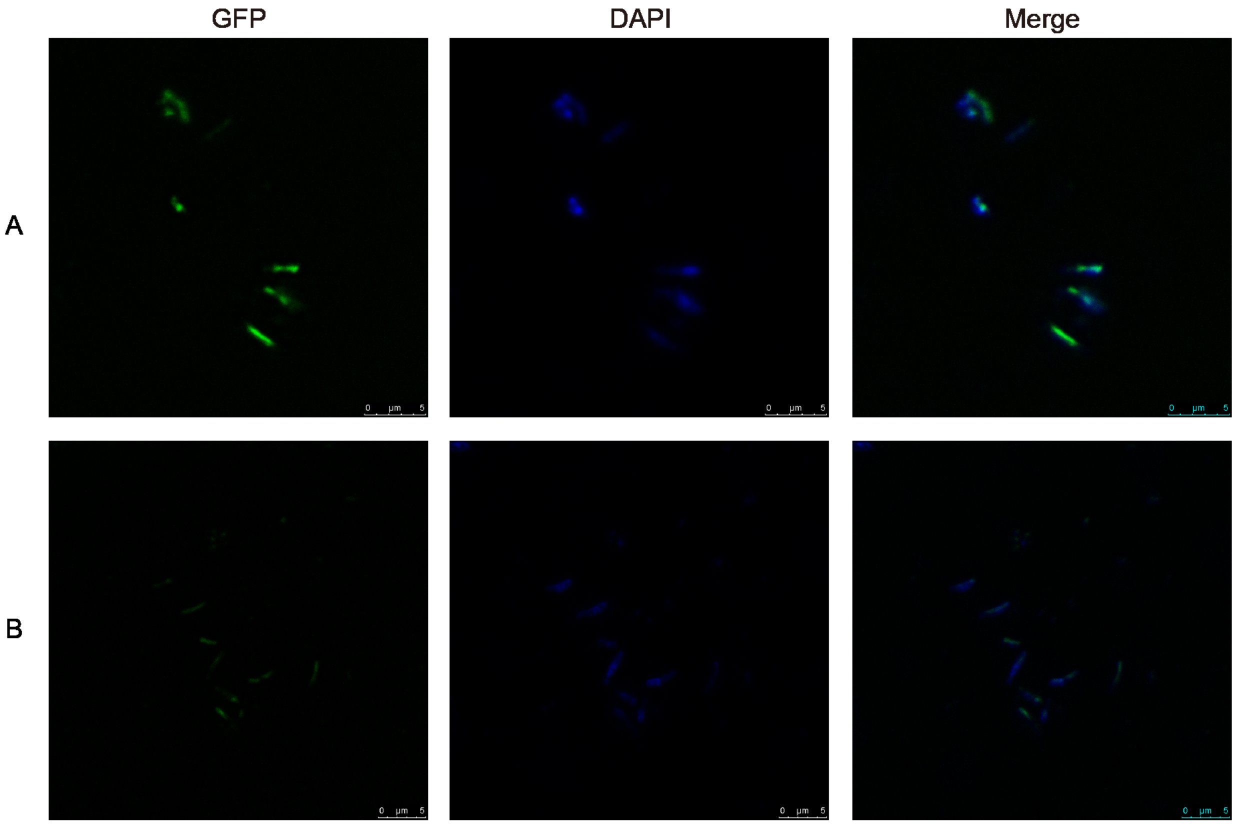

Supplemental Fig. S3. Fluorescence microscopy analysis. (A)*MsmΔ6171* strains carrying pMV261-GFP fusion protein and (B)*MsmΔ6171* strains carrying pMV261-MSMEG_6171-GFP fusion protein were analyzed by fluorescent microscopy. Bacteria were counterstained with DAPI for nuclei (blue). Scale bars, 5 μm.

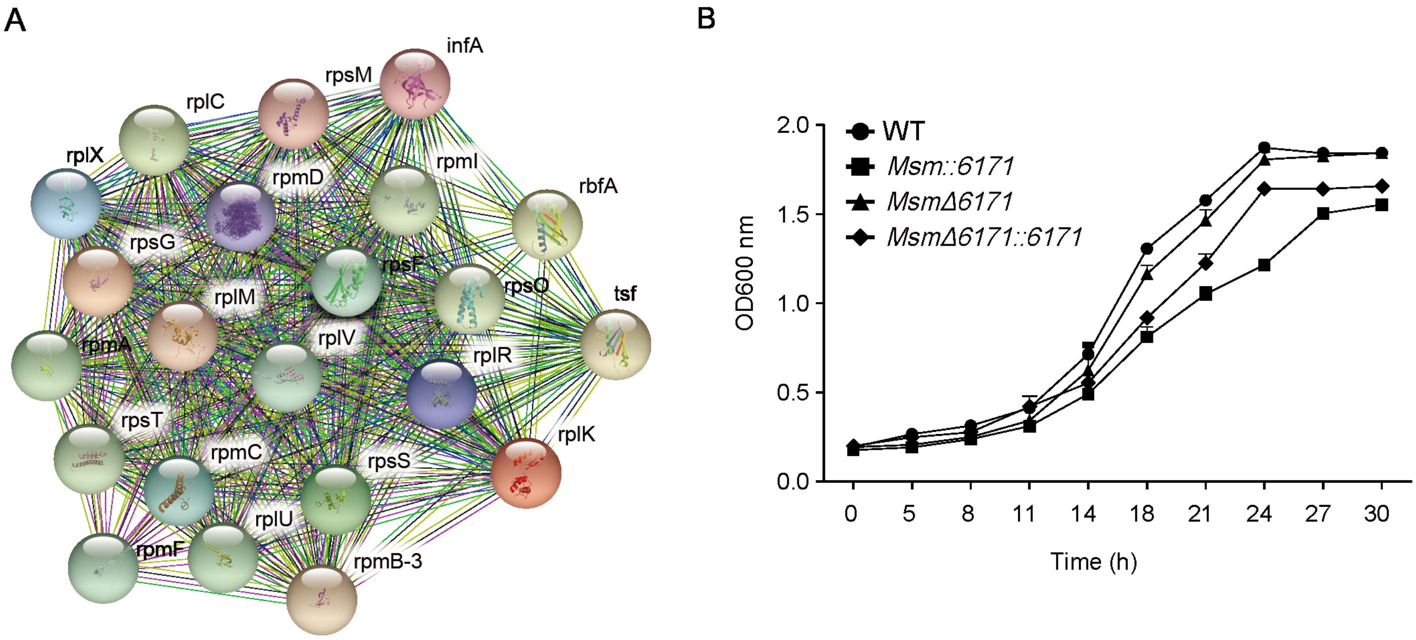

Supplemental Fig. S4. Network analysis of down-regulated proteins potentially regulated by MSMEG_6171. (A) A subnet of down-regulated proteins of *Msm::6171* recombinant strains involved in the signaling pathways of Ribosome using STRING 10.0. **(B)** Growth of recombinant Msm strains in 7H9 +ADC liquid media for 37 h. The average of two biological replicates is shown.

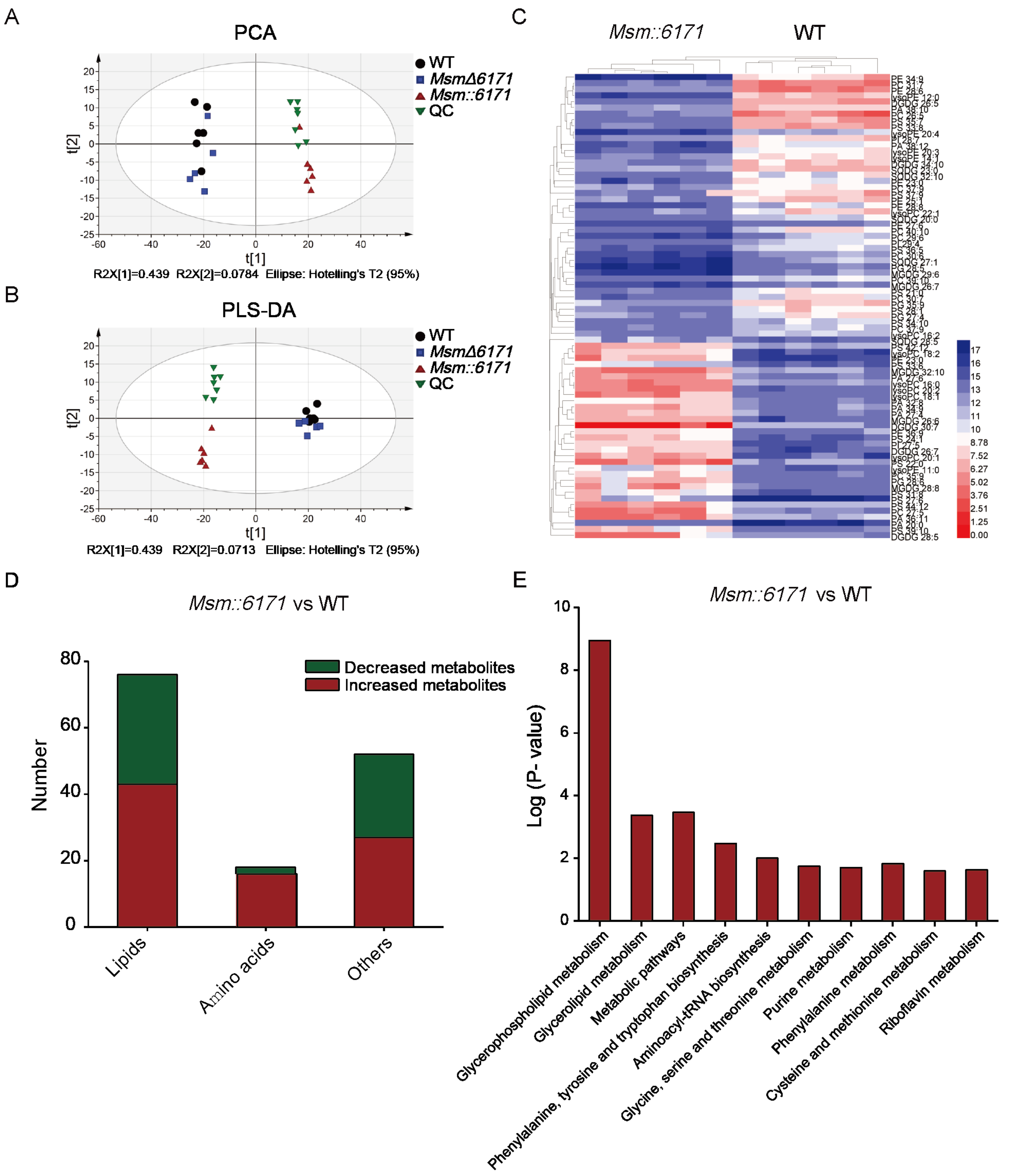

Supplemental Fig. S5. LC/MS-based metabolomics analysis of intracellular metabolites profiles. (A) PCA score and **(B)** PLS-DA score scatter plot acquired from LC-MS data in the ESI+ modes. R^2: the total explained variation of the model; Q^2: the predictability of the model. WT: wild type Msm mc^{2}155 strain, QC: quality control; **(C)** Heat map showed the relative expression level of different lipids in *Msm::6171* strain compared to WT strain. Clustering based on euclidean distance were generated using SIMCA 14.1 and featured with *P* value < 0.05. PA: phosphatidic acid, PE: phosphatidyl ethanolamine, PC: phosphatidyl choline, PS: phosphatidyl serine, PI: phosphatidyl inositol, PG: phosphatidyl glycerin, lysoPE: lysophosphatidyl ethanolamine, lysoPC: lysophosphatidyl choline, DG: diglyceride, MGDG: monogalatosyl diglyceride, DGDG: digalactosyl diglyceride, SQDG: sulfoquinovosyl diacylglycerol; **(D)** The number of significantly changed metabolites in each category of *Msm::6171* strains compared to WT strains. Red, increased metabolites; green, decreased metabolites. **(E)** Histogram displayed KEGG pathway of the significantly changed metabolites in *Msm::6171* strain according to the *P* value.
