# Supplementary material for: Integrated Quantitative Proteomics and Metabolome Profiling Reveal MSMEG_6171 Overexpression Perturbing Lipid Metabolism of Mycobacterium smegmatis Leading to Increased Vancomycin Resistance
Source: Front Microbiol. 2020 Jul 24;11:1572. doi: 10.3389/fmicb.2020.01572 (PMC7393984; doi:10.3389/fmicb.2020.01572)
Supplement: Supplementary file 2 [file Data_Sheet_2.pdf]

SUPPLEMENTAL MATERIALS AND METHODS

Bacterial strains, growth conditions and plasmid constructs

M. smegmatis mc^2155, *Escherichia coli* DH5a were used in the research. *M. smegmatis* were grown in Middlebrook 7H9 medium with 0.2% glycerol, 0.05% Tween 80 and 50 mg/mL of kanamycin where necessary and Middlebrook 7H10 medium with 2% glycerol was used for Cloning of *M. smegmatis*. *Escherichia coli* DH5a was grown in Luria-Bertani (LB) medium with 50 mg/mL of kanamycin, cells were collected in the exponential phase. DH5α/T1 Cloning Vector was cultured in LB plates supplemented with 100 μg/mL ampicillin, 40μg/mL X-Gal and 24μg/mL IPTG. The cultures were grown at 37°C with shaking of 200 rpm.

The molecular biology experiment used in the experiment was performed as described[1]. An *M. smegmatis* mc^2155 mutant without *msmeg_6171* was constructed by performing allelic exchange using *p1NIL/pGOAL19*-based flexible cassette[2]. Plasmid containing 855bp segment upstream of *msmeg_6171* + first 129bp of Msmeg_6171 (5' fragment) followed by the last 120bp of *msmeg_6171* + 849bp segment downstream of the *msmeg_6171* (3' fragment). 5' fragment was PCR amplified using the oligo 6171UF and 6171UR. The 3' fragment was PCR amplified using the oligo 6171DF and 6171DR (Supplemental Table S1). Then using 5' fragment and 3' fragment of PCR product as a template, 6171-UF and 6171-DR as prime, the PCR product was digested with *Hin*dIII and *Pac*I and cloned in *p1NIL* plasmid digested by the same restriction enzymes. The plasmid *pGOAL19* (carry the sacB, LacZ and, Hyg genes) were digested with *Pac*I and ligated to the linearized *p1NIL*, create the suicide vector. The recombinational vector was electroporated into cells of *M. smegmatis* mc^2155 strain. Blue colonies were restreaked on 7H10 plates containing hygromycin (200 mg/ml), kanamycin (25mg/ml) and X-Gal (50mg/ml). The selected cells were then resuspended in 7H9 medium, serially diluted and plated on 7H10 containing 2% sucrose and X-gal. White colonies were isolated and identified by PCR analysis. Plasmid *pMV261-MSMEG_6171* was used for complementation analysis. Recombinant plasmids were purified, confirmed, and

electroporated into the *msmeg_6171*-deleted strains, select were performed as previously described[3].

The nucleotide sequences corresponding to *msmeg_6171,* NTD and CTD of the *msmeg_6171* (*MsmΔ6171::6171*$^{Δ81-316}$*, MsmΔ6171::6171*$^{Δ1-80}$) were PCR amplified from *M. smegmatis* mc^2155 genome, gene-specific primers were as follows: 6171F / 6171R, 6171_{1-80} F / 6171_{1-80} R, 6171_{81-316} F / 6171_{81-316} R, respectively (Supplemental Table S1). PCR product and the plasmid *pMV261* were digested with *Eco*RI and *Hin*dIII, cloning into the vector *pMV261* to generate the recombinational vector (*pMV261-msmeg_6171*, *pMV261- MSMEG_6171*$_{1-80}$*, pMV261-MSMEG_6171*$_{81-316}$), then *pMV261- MSMEG _6171* was electroporated into *M. smegmatis*, *pMV261- MSMEG_6171*$_{1-80}$ and *pMV261- MSMEG_6171*$_{81-316}$ were electroporated into *MsmΔ6171* strains*,* wild type *M. smegmatis* mc^2155 was used as a control. The electroporated recombinant *M. smegmatis* cells were plated on Middlebrook 7H10 agar containing 50 µg/mL kanamycin after in vitro growth in Middlebrook 7H9 liquid medium for 3 hours. The positive cells were further verified by PCR.

Site-directed mutagenesis - The *msmeg_6171* gene was mutated by whole plasmid PCR based site-directed mutagenesis method using three pairs of specific primers $6171^{S99A,V100A}$ F / $6171^{S99A,V100A}$ R, 6171^{R249A} F / 6171^{R249}A F, $6171^{L279A,D280A}$ F / $6171^{L279A,D280A}$ R to generate 5 single point mutants where Ser99, Val100 are replaced by Ala, Ala respectively; Arg247 is replaced by Val; Leu279, Asp280 is replaced by Ala, Ala, respectively. Then using the recombinational vector *pMV261-MSMEG_6171* as a template, the PCR product was purified, confirmed by DNA sequencing, and electroporated into the *MsmΔ6171* strains, select were performed as previously described [3].

Laser Scanning Confocal Micrcopy.

1-2 ml recombinant Msm strains were cultured and pelleted by centrifugation, washed in PBS, fixed in 4% paraformaldehyde (PAF) and stored at 4℃ until further use [4]. Fluorescent staining was performed as previously described [5,6]. BODIPY-FL conjugate of vancomycin (Van-FL, Molecular Probes, Invitrogen) and

unlabelled vancomycin (sigma) each at 1 mg/ml (This was added to a final concentration of vancomycin was 2 mg/ml) were added directly to 1~2 ml Logarithmic phase culture and incubated under standard growth conditions for 90 min. Cells were collected and washed twice in 0.5 ml PBS, and finally resuspended in 50 ml PBS. Cell aliquots (10 μL) were spread on poly-L-lysine coated slides (BDH) follow on adding a drop of Prolong Antifade reagent with 100 ng/ml of DAPI (Sigma). Samples were mounted under a coverslip and examined using a Zeiss LSM880 inverted widefield micrcope, cells measured using Zen Blue 2.3 software.

Flag-based pulldown assay

The in vivo interactions for MSMEG_6171 were analyzed by Flag-based pulldown assay according to previously published procedures with some modifications. Full length *msmeg_6171* gene was cloned into the vector *pMV261* plasmid with an N-terminus 3X-Flag tag. Vector construction, screening, and confirmation were performed in *E. coli*. Completed vectors were confirmed via colony PCR and direct sequencing. Vectors were transformed into *M. smegmatis*, as described above. Transformed *M. smegmatis* was cultured on Middlebrook 7H10 agar plates (supplemented with 50 μg/ml kanamycin) for 5 days at 37°C, then cultured an additional 2 days at 25°C to allow accumulation of recombinant protein. The expression of recombinant protein was confirmed visually by anti-3X-Flag Western blot (Santa Cruz Biotechnology, Santa Cruz, CA). Samples of 150 mg of bacteria expressing the bait vectors were harvested, washed once with Hank's Buffered Salt Solution (HBSS, Invitrogen, Carlsbad, CA) and resuspended in 1.5 ml denaturing buffer (500 mM NaCl, 7 M urea, 20 mM Tris-HCl, 10 mM imidazole, .5% (v/v) glycerol, .2% (v/v) TritonX-100, 1 mM PMSF, pH 8). Bacteria were mechanically disrupted in a bead mill with 1 mm silica beads (6 cycles, 20 s each @ max speed). Samples were centrifuged at 12,000 × g for 5 min and the supernate was transferred to a clean tube. 50 μl of Ni^{2+} coated magnetic beads (Promega, Madison, WI) were added to each sample. Samples were incubated on a rotating mixer at 4°C for 2 h. After incubation, samples were washed three times in denaturing buffer. Each sample

was split into two aliquots, one for exposure to the prey lysate and the other for exposure to the control lysate, *M. smegmatis* with an unmodified pMV261 plasmid. Lysate from *M. smegmatis* carrying the *pMV261-MSMEG_6171-3X Flag* prey construct was prepared in a urea-based denaturing buffer, as described above. 1.5 ml of prey lysate was added to one aliquot of each sample containing immobilized bait protein. Proteins were allowed to refold together through sequential dilution of the denaturing buffer with HBSS. Samples were diluted by the addition of 1 ml aliquots of HBSS (pH 7.4, 10 mM imidazole), followed by 5 min incubations until the concentration of urea reached 350 mM (sample volume 30 ml). Proteins immobilized on magnetic beads were purified with a magnetic rack (Promega) and washed three times with HBSS. The beads were resuspended in laemmli buffer (+5% βbME) and proteins were separated by SDS-PAGE. Proteins were visualized with silver staining. The unique band was isolated and processed by in-gel trypsin digest before mass spectrometry analysis.

Drug susceptibility testing (MIC)

The MICs of the drugs were determined as described by Luciano Mengatto et al.[7]. To prepare the inoculum, the bacterial suspension adjusted to equal the density of a 1 McFarland standard was diluted 1:25 with 7H9 broth and 100 ml was used as an inoculum. 200 μl sterile water was added to all outer wells of sterile 96-well plates. 100 μl 7H9 broth was added in each well and serial two-fold dilutions of each drug were prepared directly on the plate by adding 100 ml of the drug working solutions. The final drug concentration ranges were 0.125 to 128 μg/ml. 100 μl of the inoculum were added to the wells. Six growth controls containing no antibiotic were included for each isolate. The plates were sealed, placed in plastic bags and incubated at 37°C at room tempreture. On day-3, 50 ml of the tetrazolium-Tween 80 mixture {1.5 ml of tetrazolium [3-(4,5-dimethylthiazol-2-yl)-2,5diphenyl-tetrazolium bromide] [ICN Biomedicals, Aurora, Oh, US] at a dilution of 1 mg/ml in absolute ethanol and 1.5 ml of 10% Tween 80 was added to one growth control and the plate was then incubated at 37°C for 12 h. If this well turned purple, the tetrazolium-Tween 80 mixture was

added to all wells and the color was recorded at 12 h. Otherwise, if the growth control well remained yellow the plate was then incubated at 37°C for 12 h, after which tetrazolium-Tween 80 mixture was added to another control before the plate was incubated for another 12 h. If this well remained yellow, incubation was continued and tetrazolium-Tween 80 solutions was added to each of the remaining four controls on days 5, 6, and 7.

Protein preparation and iTRAQ Labeling

The proteins were extracted using a mechanical crushing method[8]. Cells were resuspended in 10 mL of 50 mM Tris (pH 7.0), 150 mL NaCl and 0.5 mM phenylmethanesulphonyl fluoride (PMSF) at 4 ℃, and disrupted in a Fastprep-24 (MP Biomedicals). The resulting whole-cell extract was subjected to ultracentrifugation at 12,000 × g for 15 min using an SW 41 Ti rotor (Sigma). Supernatants were filtered twice with a 0.22 μm pore Millex filter unit (Millipore). For each sample, total protein (100 μg) was digested with 3.3 μl of trypsin (1 μg/μl) (Promega, Madison, WI, USA) at 37°C for 24 h. After trypsin digestion, peptides were dried via vacuum centrifugation. Peptides were reconstituted in 0.5 M TEAB and processed according to the manufacturer's instructions (Applied Biosystems). Briefly, one unit of iTRAQ reagent (AB Sciex, Foster City, CA, USA) was thawed and reconstituted in 24 μL Ethanol. Briefly, the control group was labeled with iTRAQ reagent 114,115,116, the experimental group was labeled with iTRAQ reagent 117,118,121. The peptides were then incubated at room temperature for 2 h. The labeled samples were then mixed and desalted with a SPEC18 ODS SPE Bulk Sorben column (Agilent, USA) and vacuum dried, providing three biological replicates for each group.

Strong cation exchange (SCX) chromatography

The iTRAQ-labeled peptide mixtures were dissolved in buffer A (NH3 in H_2O, pH=10) and loaded onto a C18 column (ACQUITY UPLC CSH, 130Å, 1.7 μm, 2.1 mm X 150 mm, 1/pkg, Waters, USA). The peptides were eluted at a flow rate of 200 μL/min with a gradient of 5-10% buffer B (80%ACN NH3 in H_2O, pH=10) for 10

min, 10-38% buffer B for 60 min and 38-95% buffer B for 10 min. A total of twelve SCX fractions were collected for one run. The system was then maintained at 5% buffer B for 30 min before the next injection. The eluted peptides vacuum dried.

The fractions above were dissolved in an aqueous solution containing 0.1% FA. Five Microliter supernatant was loaded on an EASY nLC1000 (Thermo Fisher Scientific, USA) by the autosampler onto an analytical C18 column (75 um i.d. x 150mm, 2 um, 100Å, nanoViper, Thermo Fisher Scientific, USA). The mobile phases used were composed of solvent A (0.1% FA in ACN) and solvent B (0.1% FA in H_2O). The gradient was run at 300 nL/min for 60 min at 7-35% solvent B, followed by running a linear gradient to 90% for 5 min, maintained at 90% B for 8 min. The peptides were subjected to nano-electrospray ionization followed by tandem mass spectrometry (MS/MS) in a Q EXACTIVE (Thermo Fisher Scientific, San Jose, CA, USA) coupled to the nLC. Intact peptides were detected in the Orbitrap at a resolution of 70,000 and a mass range of 350−1,800 m/z. Peptides were selected for MS/MS using high-energy collision dissociation (HCD), and ion fragments were detected in the Orbitrap at a resolution of 17,500. The electrospray voltage applied was 2.0 kV. MS/MS analysis was required for the 20 most abundant precursor ions.

Peptides and proteins were identified and quantified with Sequest HT search engine using Proteome Discoverer v2.2 (Thermo Fisher Scientific) software. A standardized iTRAQ 6 plex quantification workflow module within the Proteome Discoverer was slightly modified as below and utilized for the analysis. MS/MS data were searched against the *M. smegmatis* (strain ATCC, 700084) UniProt database. The search parameters include 10 ppm precursor mass tolerance, 0.02 Da fragment mass tolerance, and trypsin miscleavage setting of two. Static modification settings included carbamidomethylation (+57.021 Da) on cysteine and iTRAQ 6 plex (114.102 Da) on N-terminus and lysine, while dynamic modifications were set to include oxidation (+15.995 Da) on methionine and Acetyl (+42.011 Da) on protein N-Terminus. Peptide spectrum matches (PSMs) were verified based on q-values set to 1% false discovery rate (FDR) using the Percolator module. Reporter Ions Quantifier

node was used in the processing step workflow, and the Peptide and Protein Quantifier node was used in the consensus workflow of the Proteome Discoverer v2.2 to calculate and quantify peptides and protein abundances and ratios across samples. The final proteins that were deemed to be differentially expressed were filtered as a P value <0.05 and 1.5-fold changes (>1.50 or <0.667) relative to the control group.

Metabolite extraction for LC-MS

The collected Msm were washed 3 times with PBS and then quenched immediately by liquid nitrogen for 10 min stored at -80 °C. For intracellular metabolites, samples were reconstituted by dissolving in 1 mL solvent mixture containing water/methanol/acetonitrile (1 ∶2 ∶2) and disrupted in a Fastprep-24 (MP Biomedicals). The resulting whole extract was subjected to ultracentrifugation at 12,000 × g for 15 min using an SW 41 Ti rotor (Sigma). Supernatants were filtered twice with a 0.22 µm pore Millex filter unit (Millipore) and transferred to LC vial for LC–MS analysis. For lipids extraction, Chloroform/methanol solution was added to each sample. The mixture was mixed by a vortex mixer for 5 min and carried out by Ultrasonic cleaner for 10 min. Then the mixture was centrifuged at 12000 rpm, 4℃ for 10 min. The supernatant was taken in the clean test tube and the precipitates were extracted by 2 ml chloroform/methanol solution twice. All supernatant was dried by N_2 and then dissolved by chloroform/methanol (2:1, v:v). 200 µl supernatant was transferred to sampler vials for detected. As for culture supernatant, the secondary subcultures were centrifuged at 3000 rpm for 30 min to obtain the supernatant which was filtered twice using 0.22 µm filters (Millipore, Billerica, MA, USA). Supernatant were reconstituted by dissolving in 1 mL solvent mixture containing methanol/acetonitrile (1 : 1). The samples were vortexed for 30 s and -20°C for 2 h. Then centrifuged at 13 000 g for 15 min at 4 °C and transferred to LC vial for LC–MS analysis. Quality control (QC) samples were prepared by mixing each aliquot with a pooled sample and analyzing them in parallel using the same method. The QCs were injected at regular intervals (every 8 samples) throughout the analytical run to provide a set of data from which repeatability could be assessed.

Identification of metabolites by LC-MS

Intracellular metabolites - The separation was performed by Ekspert UltraLC (110, AB Sciex) and equipped with ACQUITY UPLC HSS T3 (1.8 μm 2.1×100 mm, Waters) column at a flow rate of 0.3 mL/min under a gradient program in which mobile phase A (water: acetonitrile: formic acid 900:100:1) and mobile phase B (acetonitrile: water: formic acid 900:100:1). The gradient program was applied as follows: t=4 min, 0% B; t=6 min, 25% B; t=25 min, 100% B; t=29.1 min, 100% B; t=31 min, 0% B; t=33 min, 0% B. The stop time was 40 min. For MS analysis, data were acquired by AB 5600 + Triple TOF mass spectrometer (SCIEX, Redwood City, CA, USA) operating in the positive ion mode. The capillary voltage was set at + 5500 V (positive mode). Other source conditions were kept constant in all the experiments as follows: the pressure of nebulizer gas (nitrogen) was 40 pa. The sheath gas was maintained at a temperature of 550 °C. The scan range was adjusted to 100–1200 m/z.

Lipids metabolites - Dionex UltiMate 3000 (UHPLC) -Thermo Orbitrap Elite was used for LC-MS analysis. The chromatographic column used was Waters UPLC RBEH C18 (1.7 μm 100*2.1 mm). Mobile phase: A: aqueous solution with 0.1% formic acid (0.1% 1 mmol/L NH_4COOH). B: acetonitrile/isopropanol solution (the ratio of acetonitrile and isopropanol was 1:1, 0.1% 1 mmol/L NH_4COOH, 0.1% HCOOH). Flow rate: 0.40 ml/min. Column temperature: 45℃. Injection volume: 4 μl. Gradient elution condition optimized: 0-2 min，35-80% B；2-9 min，80-100% B；9-16 min，100% B；16-20 min，100-35% B. Post time was set as 3 min for system balance. Mass spectrometry was operated in both positive and negative ion modes. The parameters optimized were as follows. Postive, heater Temp 300℃; Sheath Gas Flow rate 45 arb; Aux Gas Flow Rate 15 arb; Sweep Gas Flow Rate 1 arb; spray voltage 3.0 KV; Capillary Temp 350°C, S-Lens RF Level 30%. Mass range: m/z 200-1500。Negtive, Heater Temp 300°C; Sheath Gas Flow rate 45 arb; Aux Gas Flow Rate 15 arb; Sweep Gas Flow Rate 1 arb; spray voltage 2.5 KV; Capillary Temp 350°C, S-Lens RF Level 60%; Mass range: m/z 200-1500.

Data processing and statistical data analysis

Intracellular metabolites - The raw MS files (WIFF format file) were converted to ABF (analysis base file format) using the freely available Reifycs file converter (http://www.reifycs.com/AbfConverter/). Peak picking and alignment were performed using MS-DIAL version 2.24 and the parameters were set as follows: Alignment: MS1 tolerance, 0.01 Da; Retention time tolerance, 0.1 min; Identification: Accurate mass tolerance (MS1), 0.025 Da; Accurate mass tolerance (MS1), 0.25 Da. Representative MS/MS spectra were exported in abf format for MS-DIAL, and compound identification was performed against MS/MS libraries including MassBank[9]and MONA[10].

Lipids metabolites - Raw data were converted the common (mz.data) format by Agilent Masshunter Qualitative Analysis B.08.00 software (Agilent Technologies, USA). In the R software platform, the XCMS program was used in peak identification, retention time correction, automatic integration pretreatment. Then the data were subjected to internal standard normalization and weight normalization. Visualization matrices containing sample name, m/z-RT pair and peak area was obtained. 1801 features were got in positive mode and 636 features in negative mode. After editing, the data matrices were imported into SIMCA-P 13.0[11](Umetrics, Umea, Sweden), mean-centered and scaled to Pareto variance. Then, a multivariate analysis was conducted.

References

1. Parish T & Lewis J & Stoker NG (2001) Use of the mycobacteriophage L5 excisionase in *Mycobacterium tuberculosis* to demonstrate gene essentiality. Tuberculosis (Edinb) 81, 359-364.

2. Parish T & Stoker NG (2000) Use of a flexible cassette method to generate a double unmarked *Mycobacterium tuberculosis* tlyA plcABC mutant by gene replacement. MICROBIOLOGY+ 146 (Pt 8), 1969-1975.

3. Zanfardino A, Migliardi A, D'Alonzo D, Lombardi A, Varcamonti M & Cordone

A (2016) Inactivation of MSMEG_0412 gene drastically affects surface related properties of *Mycobacterium smegmatis*. BMC MICROBIOL 16, 267.

4. Thanky NR, Young DB, Robertson BD (2007) Unusual features of the cell cycle in mycobacteria: polar-restricted growth and the snapping-model of cell division. Tubercul is (Edinb) 87: 231: 231:

5. Daniel RA, Errington J (2003) Control of cell morphogenesis in bacteria: two distinct ways to make a rod-shaped cell. Cell 113: 767 113:

6. Joyce G, Williams KJ, Robb M, Noens E, Tizzano B, Shahrezaei V, Robertson BD (2012) Cell Division Site Placement and Asymmetric Growth in Mycobacteria. PLOS One 7(9),e44582.

7. Mengatto L & Chiani Y & Imaz MS (2006) Evaluation of rapid alternative methods for drug susceptibility testing in clinical isolates of *Mycobacterium tuberculosis*. Mem Inst Oswaldo Cruz 101, 535-542.

8. Sharma S, Rajmani RS, Kumar A, Bhaskar A, Singh A, Manivel V, Tyagi AK & Rao KV (2015) Differential proteomics approach to identify putative protective antigens of *Mycobacterium tuberculosis* presented during early stages of macrophage infection and their evaluation as DNA vaccines. INDIAN J EXP BIOL 53, 429-439.

9. Horai H, Arita M, Kanaya S, Nihei Y, Ikeda T, Suwa K, Ojima Y, Tanaka K, Tanaka S, Aoshima K, Oda Y, Kakazu Y, Kusano M, Tohge T, Matsuda F, Sawada Y, Hirai MY, Nakanishi H, Ikeda K, Akimoto N, Maoka T, Takahashi H, Ara T, Sakurai N, Suzuki H, Shibata D, Neumann S, Iida T, Tanaka K, Funatsu K, Matsuura F, Soga T, Taguchi R, Saito K & Nishioka T (2010) MassBank: a public repository for sharing mass spectral data for life sciences. J MASS SPECTROM 45, 703-714.

10. Merker M, Blin C, Mona S, Duforet-Frebourg N, Lecher S, Willery E, Blum MG, Rusch-Gerdes S, Mokrousov I, Aleksic E, Allix-Beguec C, Antierens A, Augustynowicz-Kopec E, Ballif M, Barletta F, Beck HP, Barry CR, Bonnet M, Borroni E, Campos-Herrero I, Cirillo D, Cox H, Crowe S, Crudu V, Diel R, Drobniewski F, Fauville-Dufaux M, Gagneux S, Ghebremichael S, Hanekom M, Hoffner S, Jiao WW, Kalon S, Kohl TA, Kontsevaya I, Lillebaek T, Maeda S, Nikolayevskyy V, Rasmussen M, Rastogi N, Samper S, Sanchez-Padilla E, Savic B,

Shamputa IC, Shen A, Sng LH, Stakenas P, Toit K, Varaine F, Vukovic D, Wahl C, Warren R, Supply P, Niemann S & Wirth T (2015) Evolutionary history and global spread of the *Mycobacterium tuberculosis* Beijing lineage. NAT GENET 47, 242-249.

11. Bjerrum JT, Wang Y, Hao F, Coskun M, Ludwig C, Gunther U & Nielsen OH (2015) Metabonomics of human fecal extracts characterize ulcerative colitis, Crohn's disease and healthy individuals. METABOLOMICS 11, 122-133.
